# Machine learning models predict coagulopathy in spontaneous intracerebral hemorrhage patients in ER

**DOI:** 10.1111/cns.13509

**Published:** 2020-11-28

**Authors:** Fengping Zhu, Zhiguang Pan, Ying Tang, Pengfei Fu, Sijie Cheng, Wenzhong Hou, Qi Zhang, Hong Huang, Yirui Sun

**Affiliations:** ^1^ Department of Neurosurgery Huahsan Hospital Fudan University Shanghai China; ^2^ Neurosurgical Institute of Fudan University Shanghai China; ^3^ Shanghai Clinical Medical Center of Neurosurgery Shanghai China; ^4^ Shanghai Key Laboratory of Brain Function and Restoration and Neural Regeneration Shanghai China; ^5^ Department of Nursing Huahsan Hospital Fudan University Shanghai China; ^6^ Information Center Huahsan Hospital Fudan University Shanghai China

**Keywords:** coagulopathy, intracranial hemorrhage, machine learning, random forest, support vector machine

## Abstract

**Aims:**

Coagulation abnormality is one of the primary concerns for patients with spontaneous intracerebral hemorrhage admitted to ER. Conventional laboratory indicators require hours for coagulopathy diagnosis, which brings difficulties for appropriate intervention within the optimal window. This study evaluates the possibility of building efficient coagulopathy prediction models using data mining and machine learning algorithms.

**Methods:**

A retrospective cohort enrolled 1668 cases with acute spontaneous intracerebral hemorrhage from three medical centers, excluding those under antithrombotic therapies. Coagulopathy‐related clinical parameters were initially screened by univariate analysis. Two machine learning algorithms, the random forest and the support vector machine, were deployed via an approach of four‐fold cross‐validation to screen out the most important parameters contributing to the occurrence of coagulopathy. Model discrimination was assessed using metrics, including accuracy, precision, recall, and F1 score.

**Results:**

Albumin/globulin ratio, neutrophil count, lymphocyte percentage, aspartate transaminase, alanine transaminase, hemoglobin, platelet count, white blood cell count, neutrophil percentage, systolic and diastolic pressure were identified as major predictors to the occurrence of acute coagulopathy. Compared to support vector machine, the model based on the random forest algorithm showed better accuracy (93.1%, 95% confidence interval [CI]: 0.913‐0.950), precision (92.4%, 95% CI: 0.897‐0.951), F1 score (91.5%, 95% CI: 0.889‐0.964), and recall score (93.6%, 95% CI: 0.909‐0.964), and yielded higher area under the receiver operating characteristic curve (AU‐ROC) (0.962, 95% CI: 0.942‐0.982).

**Conclusion:**

The constructed models exhibit good prediction accuracy and efficiency. It might be used in clinical practice to facilitate target intervention for acute coagulopathy in patients with spontaneous intracerebral hemorrhage.

## Graphical abstract

Coagulopathy is a common and severe complication of spontaneous intracerebral hemorrhage and significantly affects patients’ outcome and may offer an early warning and facilitate ancillary resource management of patients admitted to the Emergency Department.

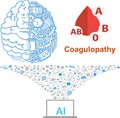

## 1 INTRODUCTION

Spontaneous intracerebral hemorrhage (ICH) is a major global public health issue, contributing to 7.4 million cases and over 3.1 million deaths worldwide annually.^1^ The case‐fatality rate of ICH ranges from 35% at seven days to 59% at one year.^2^, ^3^, ^4^ Survivors often present with severe disability, leaving less than 40% regaining functional independence.^4^

It has been established that prolonged bleeding frequently occurs during the acute phase of ICH, which contributes to neurological deterioration and worsened outcome.^3^, ^5^ The pathogenesis of prolonged bleeding is not fully understood, but coagulation abnormalities are considered as one of the most significant risk factors.^3^, ^6^ For patients under antithrombotic therapies (including antiplatelet, anticoagulant, and fibrinolytic agents), the application of hemostatic agents such as vitamin K_1_ is a common regimen in neurological ICU and is recommended by ICH guidelines from the American Heart Association and the American Stroke Association.^4^ Even without any antithrombotic therapies, coagulation abnormalities are common and are often accompanied by intracerebral hematoma enlargement.^7^ Early detection and intervention of acute coagulopathy can significantly reduce mortality and improve outcomes.^8^, ^9^

Currently, the diagnosis of coagulopathy is based on conventional coagulation indicators such as prothrombin time (PT), international normalized ratio (INR), and activated partial thromboplastin time (APTT), which typically require a minimum of 1 to 2 hours of processing time after blood sample collection. A most optimal therapeutic window may be missed due to this time lag. Therefore, it is of great importance to develop efficient prediction models that can identify coagulopathy rapidly and timely, so as to provide an early warning to physicians and to facilitate ancillary resource management better to treat patients in the emergency room (ER).

Previously, clinical parameters, including age, gender, body temperature, have been identified as risk factors for coagulation abnormalities in acute ICH patients, yet the sensitivities and specificities varied among studies.^10^, ^11^, ^12^, ^13^, ^14^, ^15^ The difficulties of precise predictions come from the fact that the coagulopathy during the acute phase of ICH is a multifactorial pathological process with complex mechanisms, possibly including tissue damage, hypoxemia, acidemia, inflammation, hypoperfusion, and other confounders. For ICH patients arriving at the ER, it is impractical for clinicians to screen every individual factor. Therefore, it is crucial to develop prediction models that are easily applicable to alert clinicians of potential coagulopathy in ICH patients.

The rise of big data analysis and machine learning algorithms offers possible strategies to build efficient prediction models and reveal hidden patterns from enormous datasets.^16^, ^17^, ^18^, ^19^, ^20^ In this study, we used machine learning methods to develop and validate a prediction model for coagulopathy after acute ICH based on objective indicators, which are routinely obtained after patients being admitted to the ER.

## 2 METHODS

### 2.1 Study cohort and source of data

The study was approved by the institutional ethics committees and registered with the Chinese Clinical Trial Registry (ChiCTR1900021522). The clinical data collected for modeling were retrospectively obtained from patients in the ER of Shanghai Huashan Hospital, Shanghai Jing'an Hospital, and Huashan Pudong Hospital between January 2016 and June 2019. The data set includes demographic information, triage target, medical records, vital signs, laboratory tests, imaging examinations, and transfusion records. Information was collected electronically from the hospital information system (HIS) and Laboratory Information Management System (LIS). Due to the retrospective nature of the study, informed consent was waived. The personal information and privacy of patients involved in this study are strictly protected under the supervision of the ethics committee.

Patients with non‐aneurysmal spontaneous ICH, confirmed by Computed Tomography (CT) scanning, were recruited in this study. The inclusion criteria also involve a timing of less than 12 hours from symptom onset to ER admission.^21^, ^22^ Since two of the medical centers, Huashan Hospital and Huashan Pudong Hospital, only accept adult patients, this study only recruited patients over 18 years old. The exclusion criteria involve any of the following conditions: pregnancy; uncorrected shock (systolic pressure ≤90 mm Hg, or diastolic pressure ≤50 mm Hg); thrombocytopenia; cirrhosis; hypohepatia/hepatic failure; renal failure; currently on oral administration of antiplatelets or anticoagulants (including warfarin, clopidogrel, aspirin, rivaroxaban, and dabigatran); and patients with incomplete data upon ER admission.

### 2.2 Definition of coagulation abnormalities

Results of coagulation assessment were collected from day 0 to 5 after ER admission. A diagnosis of coagulation abnormality was made when a patient showed either increased International Normalized Ratio (INR ≥1.2) or prolonged activated partial thromboplastin time (APTT, reference range 28‐34 seconds). These indicators and reference ranges are used to assess either the extrinsic or intrinsic pathways of coagulation along with the common pathways, which have been commonly applied within clinical researches and literature.^23^, ^24^, ^25^, ^26^

### 2.3 Statistics

Continuous variables were presented as mean ± standard deviation for variables with a normal distribution, or median and interquartile ranges for skewed distribution variables. Categorical variables were presented as proportions. Shapiro‐Wilk W test and F test were performed for normality and homogeneity of variance. The baseline characteristics between ICH patients with or without coagulopathy were initially compared using either Student's *t* tests or rank‐sum tests. The *χ* ^2^ tests or Fisher exact tests were used to compare differences in categorical variables, as appropriate. Statistical analyses were performed using SPSS 23.0 software.

### 2.4 Machine learning algorithms

The data mining and model fitting were performed in python 3.6. Two algorithms, random forest and support vector machine (SVM), were applied to predict coagulopathy among ICH patients admitted to the ER.

Random forest is a supervised learning algorithm. The "forest" in this approach is a series of decision trees that act as "weak" classifiers, which are poor predictors individually but exhibit robust prediction value in the aggregate. To classify an object from an input vector, each tree gives a classification. The forest selects the classification that has the most votes.^27^, ^28^ In this study, the Gini Index was applied as the optimization criterion, with 1000 estimators used in the calculation. The hyperparameters used in the current study were as follows: criterion=“gini,” bootstrap = True, max_parameter=“auto,” max_depth = 10, n_jobs = 2, min_samples_split = 2.

SVM, also known as support vector network (SVN), is also a supervised learning method. SVM looks at data and sorts it into one of two categories. It is trained with a series of data already classified into two groups, building the model as it is initially trained. The task of an SVM algorithm is to determine which category a new data point belongs to. This makes SVM a kind of non‐binary linear classifier.^29^, ^30^ In this study, the prediction model was trained via linear SVM, in which relative parameter contributions were derived from the weighted coefficients. To ensure the robustness of the parameter contributions, 1,000 bootstrapped sets were generated, in which 75% of the training set was sampled with replacement. Linear SVMs were trained on each of these bootstrapped sets.

This study adopts an approach of fourfold cross‐validation via which the whole data set was randomly divided into four subsets (folds). Of the fourfolds, threefolds were used as training data, and the remaining one was retained as a validation data set. The cross‐validation process was repeated four times, and each of the fourfolds was used once as validation data. The four results were then averaged to produce a single estimation. The area under the receiver operating characteristic curve (AU‐ROC), precision, classification accuracy, recall score, and F1 score were used to evaluate prediction models. Precision quantifies the percentage of positive class predictions that truly belongs to the positive class. Classification accuracy is the proportion of the correct prediction in all prediction results. The recall score is the proportion of predicted positive samples in all true positive samples. The F1 score provides a single score that balances both the concerns of precision and recall in one number.

## 3 RESULTS

### 3.1 Cohort Characteristics

**FIGURE 1:**
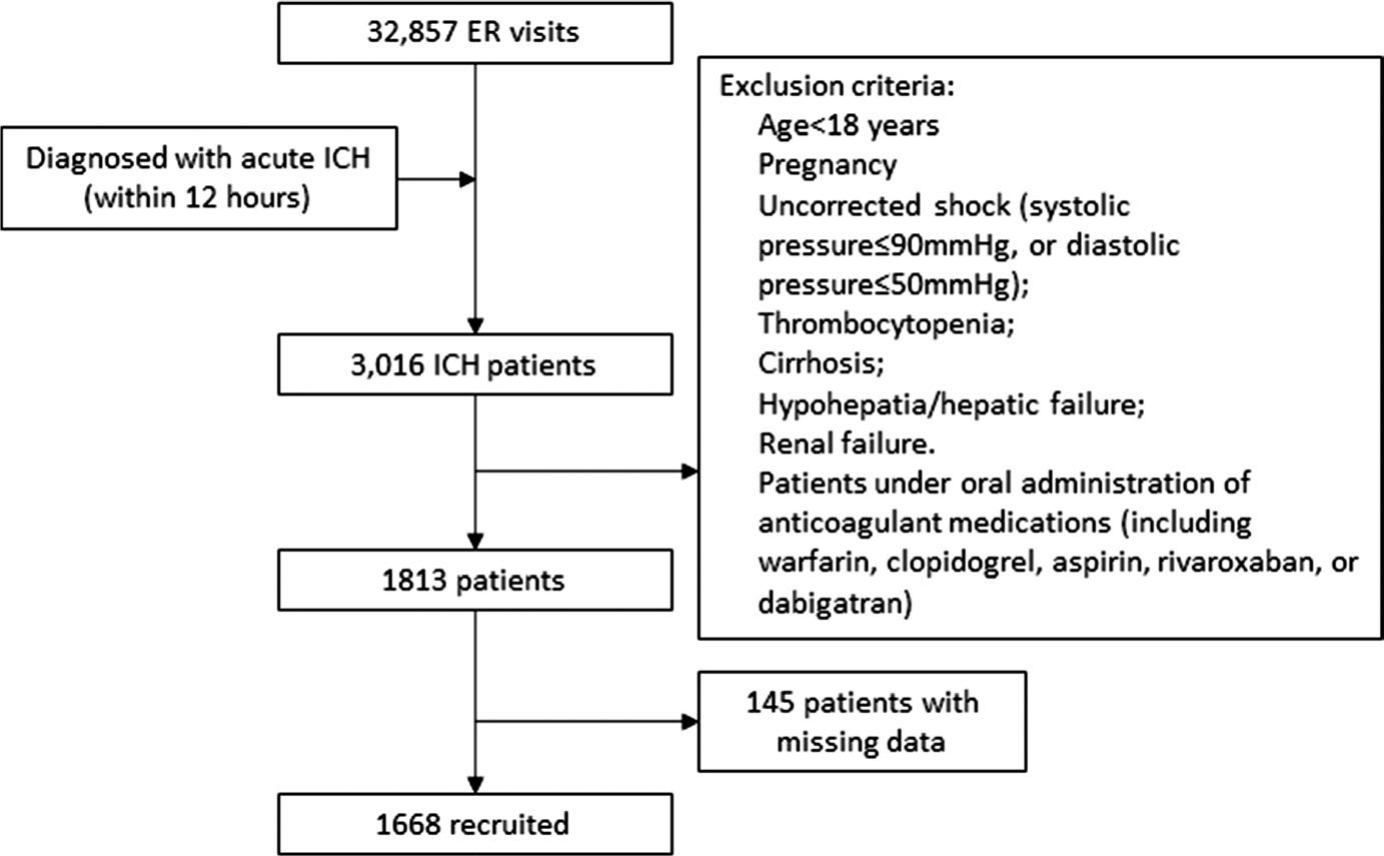
A flowchart of patient selection

Between January 2016 and June 2019, 32,857 patients visited the ER of the above three medical centers. Among them, 3,016 patients were diagnosed with acute ICH, and 1,813 patients met the above inclusion criteria. Yet, 145 patients were excluded due to missing data for one or more covariates. Therefore, data of 1,668 patients were finally used for modeling. A flowchart of patient selection is shown in Figure 1. The demographic characteristics of recruited patients are shown in Table 1.

**TABLE 1:** Characteristic of ICH patients with coagulopathy and without coagulopathy

	Overall (n = 1668)	With Coagulopathy (n = 355)	Without Coagulopathy (n = 1313)	*P*‐value*
Age	54.56 ± 15.94	54.47 ± 15.61	54.59 ± 15.72	.90
Gender (Male: Female)	943:725(1.30:1)	201:154 (1.31:1)	742:571 (1.30:1)	.95
Diagnosis n, (%)				
Lobe hemorrhage	461 (27.64)	99 (27.89)	362 (27.57)	.91
Basal ganglia hemorrhage	743 (44.54)	151 (42.54)	592 (45.09)	.39
Cerebellar hemorrhage	79 (4.74)	14 (3.94)	65 (4.95)	.48
Brain stem hemorrhage	81 (4.86)	19 (5.35)	62 (4.72)	.68
Ventricular hemorrhage	304 (18.23)	72 (20.28)	232 (17.67)	.26
Medical history n, (%)				
Hypertension	738 (44.24)	162 (45.63)	576 (43.87)	.55
Diabetes	426 (25.54)	91 (25.63)	348 (25.51)	.57
Vital sign on ER admission				
Temperature, ℃	36.93 ± 0.74	36.94 ± 0.72	36.92 ± 1.02	.73
Heart rate	90.53 ± 17.76	91.12 ± 18.23	89.57 ± 17.58	.14
Systolic blood pressure, mm Hg	132.53 ± 26.32	137.53 ± 25.59	129.82 ± 29.21	**<.01**
Diastolic blood pressure, mm Hg	78.12 ± 14.37	81.58 ± 16.17	76.38 ± 15.98	**<.01**
SpO2, %	97.32 ± 8.21	97.01 ± 8.15	97.65 ± 7.37	.16
Routine blood test				
Hb, g/L	123.24 ± 19.34	121.46 ± 17.35	126.48 ± 19.86	**<.01**
WBC, × 109/L	12.68 ± 3.24	13.64 ± 3.59	12.16 ± 3.89	**<.01**
NEUT, × 109/L	10.91 ± 2.14	11.28 ± 2.43	10.77 ± 2.96	**.029**
NEUT percentage, %	86.21 ± 12.45	87.13 ± 13.31	85.32 ± 12.64	**.026**
PLT, × 109/L	190.32 ± 24.38	177.48 ± 25.37	210.27 ± 28.24	**<.01**
LYMPH, %	11.02 ± 2.03	10.95 ± 2.21	12.37 ± 3.15	**<.01**
MPV, fL	12.41 ± 1.38	12.42 ± 1.42	12.39 ± 1.26	.89
PDW, %	16.67 ± 3.95	16.62 ± 3.82	16.75 ± 4.13	.59
Liver & renal function				
ALT, U/L	22.37 ± 7.32	23.59 ± 7.48	21.57 ± 6.96	**<.01**
AST, U/L	32.15 ± 8.35	29.35 ± 8.46	33.18 ± 9.73	**<.01**
A/G	1.64 ± 0.42	1.31 ± 0.31	1.79 ± 0.45	**<.01**
Cr, μmol/L	65.25 ± 15.21	66.34 ± 16.37	64.78 ± 17.02	.12
BUN, mmol/L	4.78 ± 1.63	4.67 ± 1.57	4.83 ± 1.72	.11
Electrolyte				
Na+, mmol/L	137.32 ± 12.30	136.77 ± 13. 41	138.33 ± 13.22	.092
K+, mmol/L	3.96 ± 1.53	3.89 ± 1.55	4.01 ± 1.54	.19
Ca2+, mmol/L	1.97 ± 0.21	1.98 ± 0.23	1.96 ± 0.22	.13
Cl‐, mmol/L	103.36 ± 11.35	102.96 ± 11.24	104.25 ± 12.87	.086

Note

Bold values indicate p‐values less than 0.05.

* The *P*‐values indicate the difference in ICH patients with and without coagulopathy. A *P*‐value <.05 is considered as statistical significance.

A total number of 1,313 (78.71%) patients did not present coagulopathy during the acute phase of ICH, while 355 (21.29%) patients had coagulation abnormalities upon ER admission. Patients with coagulopathy showed statistically higher values for systolic blood pressure (SBP) and diastolic blood pressure (DBP) (Table 1). Other parameters associated with coagulopathy include albumin/globulin ratio (A/G), neutrophil count (NEUT), lymphocyte percentage (LYMPH), aspartate transaminase (AST), alanine transaminase (ALT), hemoglobin (Hb), platelets count (PLT), white blood cell count (WBC), and neutrophil percentage in acute ICH patients. On the contrary, parameters such as age, gender, hemorrhage locations, history of hypertension, body temperature, heart rate, and pulse oxygen saturation (SpO_2_) showed no significant difference between the two groups (Table 1).

### 3.2 Random forest model

In the random forest model, recruited patients were classified according to their coagulation status, and an algorithm was used to assess the importance of each clinical parameter on coagulopathy. Parameter importance was calculated as the sum of the decrease in error when split by a variable. The importance of each clinical parameter reflects the contribution of each variable in the patient's classification into the coagulopathy or non‐coagulopathy group. Major indicators for coagulopathy during acute phase ICH were ranked in the upper part of Table 2 in the random forest model: A/G (0.232), NEUT (0.176), LYMPH (0.170), AST (0.160), Hb (0.153), PLT (0.146), WBC (0.139), NEUT percentage (0.138), SBP (0.136), and DBP (0.134).

**TABLE 2:** Feature contributions derived from machine learning algorithms

Model	Variable	Contribution^c^
Random forest		
	A/G^a^	0.232
	NEUT^b^	0.176
	LYMPH ^a^	0.170
	AST^b^	0.160
	Hb^a^	0.153
	PLT^a^	0.146
	WBC^b^	0.139
	NEUT percentage^b^	0.138
	SBP^b^	0.136
	DBP^b^	0.134
SVM		
	LYMPH^a^	0.203
	NEUT ^b^	0.199
	A/G^a^	0.187
	AST^b^	0.160
	WBC^b^	0.180
	Hb^a^	0.158
	NEUT percentage^b^	0.154
	PLT^a^	0.151
	SBP^b^	0.148
	DBP^b^	0.133

^a^ Measurements below the normal range.

^b^ Measurements above the normal range.

^c^ The values were transformed to a scale of 0‐1.

### 3.3 SVM model

Further interrogation of the linear SVM model identified the major predictor variables contributing to the presence of coagulopathy of ICH patients upon ER admission. Variables are listed in descending order of contribution in Table 2 (lower part), with higher parameter contribution denoting a stronger association with coagulopathy: LYMPH (0.203), NEUT (0.199), A/G (0.187), AST (0.160), WBC (0.180), Hb (0.158), NEUT percentage (0.154), PLT (0.151), SBP (0.148), and DBP (0.133).

### 3.4 Model performance comparison

Model discrimination was assessed using machine learning evaluation metrics, including accuracy, precision, recall, and F1 score. The results are presented in Table 3. Notably, the random forest model is superior to the SVM model in both accuracy (0.931) and precision (0.924), as well as F1 (0.915) and recall scores (0.936). The AU‐ROC values with 95% CI were 0.9623 (95% CI: 0.9415 ‐ 0.9815) for the random forest model and 0.8987 (95% CI: 0.8559 ‐ 0.9436) for SVM model.

**TABLE 3:** The performance metrics of machine learning with 95%CI

Models	Accuracy	Precision	FI score	Recall	AU‐ROC
Random forest	0.931 (0.913‐0.950)	0.924 (0.897‐0.951)	0.915 (0.889‐0.942)	0.936 (0.909‐0.964)	0.962 (0.942‐0.982)
SVM	0.902 (0.884‐0.920)	0.899 (0.872‐0.916	0.889 (0.863‐0.916)	0.911 (0.884‐0.938)	0.899 (0.856‐0.944)

### 3.5 Contributors for the extrinsic and intrinsic coagulation pathway

Since INR and APTT measure the extrinsic and intrinsic coagulation pathway, respectively, the major contributors for either of the pathway were screened independently using the same machine learning strategies. Table 4 shows that the random forest algorithm has superior accuracy, precision, FI score, and Recall Score during the prediction of both pathways. The SVM algorithm offers a higher AU‐ROC value 0.965(95% CI 0.916‐0.987) for increased INR prediction but a lower AU‐ROC value 0.921(95% CI 0.896‐0.976) for prolonged APTT prediction compared to the random forest algorithm. The variables with a significant contribution to both pathways are sorted and listed in Table 5.

**TABLE 4:** The performance metrics of machine learning with 95%CI for the extrinsic and intrinsic coagulation pathway

	Prolonged INR		Prolonged APTT	
	Random forest	SVM	Random forest	SVM
Accuracy	0.978 (0.939‐0.994)	0.973 (0.938‐0.991)	0.974 (0.936‐0.997)	0.894 (0.842‐0.934)
Precision	0.913 (0.886‐0.949)	0.889 (0.861‐0.913	0.921 (0.893‐0.958)	0.875 (0.851‐0.893)
FI score	0.917 (0.892‐0.949)	0.901 (0.871‐0.927)	0.919 (0.875‐0.953)	0.876 (0.842‐0.891)
Recall	0.925 (0.892‐0.954)	0.921 (0.891‐0.948)	0.931 (0.907‐0.961)	0.929 (0.894‐0.952)
AU‐ROC	0.942 (0.901‐0.976)	0.965 (0.916‐0.987)	0.978 (0.956‐0.992)	0.921 (0.896‐0.976)

**TABLE 5:** Feature contributions of prolonged INR or APTT derived from the better machine learning algorithm (Random Forest)

Rank	Prolonged INR		Prolonged APTT	
	Variable	Contribution^c^	Variable	Contribution^c^
1	A/G^a^	0.231	A/G^a^	0.197
2	NEUT^b^	0.191	NEUT^b^	0.162
3	LYMPH ^a^	0.148	WBC^b^	0.158
4	AST^b^	0.144	AST^b^	0.149
5	Hb^a^	0.142	LYMPH ^a^	0.144
6	ALT^b^	0.139	ALB^a^	0.142
7	ALB^a^	0.139	PLT^a^	0.139
8	NEUT percentage^b^	0.137	BUN^b^	0.138
9	PLT^a^	0.135	NEUT Percentage^b^	0.138
10	SBP^b^	0.134	ALT^b^	0.136

^a^ Measurements below the normal range.

^b^ Measurements above the normal range.

^c^ The values were transformed to a scale of 0‐1.

## 4 DISCUSSION

Coagulation is one of the most sophisticated systems of the human body, which is affected by multiple organ functions, disorders, and medications. Early detection and intervention of coagulopathy are known to reduce mortality and to improve the outcomes of ICH patients.^8^, ^9^ It is of great importance to develop effective prediction models that can alert clinicians to potential coagulopathy cases in ICH patients in the ER. Considering the narrow optimal therapeutic window for coagulation abnormalities, an ideal prediction model would be: (a) Easy to use: the input data for the prediction model should be easily accessible upon ER admission (ideally, no extra work for clinicians such as filling in questionnaires or performing scaling). (b) Timely: clinical parameters required can be quickly obtained, enabling timely prediction and appropriate model‐guided interventions. (c) Universal: clinical parameters needed for modeling should be widely applied to most medical institutions worldwide, both in developed and under‐developed areas.

Currently, ongoing investigations aim to look for practical and convenient tools for coagulopathy prediction. Machine learning techniques are extraordinary in terms of information analyzing capabilities and unveiling the hidden relationships among various parameters. Not only have they been used for weighted parameter selection, but they also help construct high predictive and optimal fitting models in various medical investigations, including neurological disorders such as intracranial stenosis^31^ and Parkinson's disease.^32^ Relying on the powerful capabilities of data selection, machine learning techniques reduce the redundant attributes of the patients' characteristics and meanwhile provide a comprehensive overview of interrelated attributes.^20^, ^33^, ^34^, ^35^ In this study, two different data mining approaches, the random forest and SVM, were constructed and proposed to predict coagulopathy among patients with acute ICH. Clinical data on epidemiological characteristics, vital signs, routine blood tests, liver and renal functions, and electrolytes were collected and used for machine learning algorithms. Both models yielded satisfactory predictive performance, and each produced an optimal combination of parameters as a predictive model. The random forest model is better in terms of accuracy, precision, F1 score, recall scores, and offered superior predicted performance in AU‐ROC, though.

In the present study, both random forest and SVM algorithms indicated that A/G, NEUT, LYMPH, and AST changes were the major predictors for the development of coagulopathy. The contributions of the A/G ratio and AST suggest that regular liver function plays a vital role in maintaining normal coagulation. Hemostasis is closely related to liver function, as most coagulation factors are synthesized by liver parenchymal cells. Also, the liver's reticuloendothelial system plays a critical role in the clearance of the activated form of the coagulation factors. The severity of coagulation abnormalities correlated to the extend of liver disturbance.^36^ Yet, bilirubin is not identified as a primary parameter for coagulopathy in this study, suggesting that synthesis dysfunction may play a more critical role than hepatocellular damage or biliary obstruction in coagulopathy.

Acute leukocytosis is a well‐established response to ICH. Previous prospective studies have shown that elevated admission WBC count and neutrophil count are associated with an increased risk of early neurologic deterioration in ICH ^37^, ^38^, ^39^ as well as in ischemic stroke.^40^ Multiple studies have also reported increased neutrophil‐to‐lymphocyte ratio associated with higher mortality and increased intracerebral remote diffusion‐weighted imaging lesions in ICH,^41^, ^42^ and worsened prognosis in glioma.^43^ Although the mechanisms are not fully understood, some interactions between coagulation factors and neutrophils are described elsewhere, which may, in turn, play a role in hemostasis. Proteins of the coagulation system, such as FXa, thrombin, and fibrinogen, bind to various sites on neutrophils. This binding leads to complicated consequences. First, it assembles coagulation complexes such as the prothrombinase complex and the contact system on the neutrophil membrane, which further impacts neutrophil functions such as chemotaxis, aggregation, degranulation, and migration. Second, neutrophil elastase degrades multiple coagulation proteins, modulating both the thrombotic and the fibrinolytic systems.^44^ In fact, these interactions are recognized as a link between the coagulation and inflammation pathways.

Multiple prospective investigations have indicated that achieving early and stable blood pressure seems safe and associated with favorable outcomes in acute ICH patients.^45^, ^46^ While this may be due to avoidance of hypertension‐induced hematoma enlargement,^47^ studies have revealed that patients with a history of hypertension show lower‐grade fibrin formation and higher levels of several anticoagulant factors (eg, antithrombin III, protein C and protein S, and von Willebrand factor antigen).^48^, ^49^ The clinical application of these findings warrants additional studies.

In this present study, the two machine learning algorithms make it convenient to achieve predicted probability for coagulopathy among patients with acute ICH, which is more efficient than conventional coagulation lab tests. Compared to traditional statistical methods, the random forest and SVM are better at analyzing nonlinear relationships between various biochemical markers and coagulopathy. Notably, the strategy of machine learning models is highly practical. All parameters used in this study are easily accessible and well established in routine clinical work. From a scientific point of view, machine learning and big data techniques have the capability of screening a vast number of candidates with high efficiency and revealing hidden patterns that could easily be overlooked during routine clinical work. From a clinical point of view, by identifying patients with primary risk factors, ER clinicians could be warned for the occurrence of coagulopathy and provide prophylactic treatment if necessary. A preliminary ER risk prediction system based on machine learning algorithms involved in this study has been under test in our facility. A demo system can be reviewed at http://52.83.50.248:9090/rpas/page/index.jsp (username: user1; password: 111111). The manuscript of introducing this system is under preparation. It is hoped that in the near future, machine learning and artificial intelligence technologies would be fully applied to assisted diagnosis, risk assessment, and treatment strategy formulation.

## 5 CONCLUSION

Machine learning techniques have been successfully introduced into the field of healthcare. This study provides an example of a systematic analysis of the data set on coagulopathy among ICH patients. The results above demonstrate that machine learning techniques can generate prediction models with excellent performance and high efficiency. Such methods and theorems could be applied to other evaluations in the future.

## CONFLICT OF INTEREST

The authors report no conflict of interest concerning the materials or methods used in this study or the findings specified in this paper.

## Data Availability

The data that support the findings of this study are available from the corresponding author upon reasonable request.
